# Ataxia Prevalence in Primary Orthostatic Tremor

**DOI:** 10.5334/tohm.570

**Published:** 2020-12-16

**Authors:** Rebecca Thompson, Danish E. Bhatti, Amy Hellman, Sarah J. Doss, Kalyan Malgireddy, James Shou, Channaiah Srikanth-Mysore, Sunil Bendi, John M. Bertoni, Diego Torres-Russotto

**Affiliations:** 1 University of Nebraska Medical Center, Department of Neurological Sciences, Nebraska Medical Center, Omaha, NE, US; 2 Dartmouth-Hitchcock Medical Center, Lebanon, New Hampshire, US

**Keywords:** orthostatic tremor, ataxia, coordination, cerebellum, tremor, balance

## Abstract

**Background:**

The exact pathophysiology of primary Orthostatic Tremor (OT) is unknown. A central oscillator is assumed, and previous imaging studies show involvement of cerebellar pathways. However, the presence of ataxia on clinical exam is disputed. We set out to study ataxia in OT prospectively.

**Methods:**

EMG-confirmed primary OT subjects and spousal controls received a neurological exam with additional semiquantitative evaluations of ataxia as part of a multinational, prospective study. These included detailed limb coordination (DLC), detailed stance and gait evaluation (DS), and the Brief Ataxia Rating Scale (BARS). Intra- and inter-rater reliability were assessed and satisfactory.

**Results:**

34 OT subjects (mean age = 67 years, 88% female) and 21 controls (mean age = 66 years, 65% male) were enrolled. Average disease duration was 18 years (range 4–44). BARS items were abnormal in 88% of OT patients. The OT subjects were more likely to have appendicular and truncal ataxia with significant differences in DLC, DS and BARS. Ocular ataxia and dysarthria were not statistically different between the groups.

**Discussion:**

Mild-to-moderate ataxia could be more common in OT than previously thought. This is supportive of cerebellar involvement in the pathophysiology of OT. We discuss possible implications for clinical care and future research.

**Highlights:**

- Previous studies of Primary Orthostatic Tremor (OT) have proposed pathophysiologic involvement of the cerebellar pathways.
- However, presence of ataxia has not been systematically studied in OT.
- This is a prospective comprehensive ataxia assessment in OT compared to controls. Mild-to-moderate appendiculo-truncal ataxia was found to be common in OT.

## Introduction

Orthostatic Tremor (OT) is a movement disorder characterized by a sensation of unsteadiness and high frequency leg tremors present only upon standing. Both the sensation of unsteadiness and the tremors disappear or improve substantially by sitting, walking or leaning [1]. The 1998 Movement Disorder Society Consensus Statement on Tremors noted that in OT clinical findings were limited to barely visible or only palpable fine amplitude leg tremors, with diagnosis requiring surface EMG (sEMG) showing a 13–18 Hz pattern [2]. These criteria were also confirmed within the 2018 Consensus Statement of the Classification of Tremors, were Primary OT was described as an *isolated* tremor syndrome devoid of other clinical findings [3]. Therefore, as an isolated tremor disorder, associated movement phenomenology (including ataxia) is not expected within OT. Primary Orthostatic Tremor Plus occurs when patients with a baseline neurological condition (e.g. Parkinson Disease or Dementia) present with an associated Orthostatic Tremor [3].

The pathophysiology of OT is not well understood. However, many factors seem to point to cerebellar involvement, starting with findings in functional imaging and the fact that some ataxic illnesses are associated with secondary OT [4]. Interestingly, the medications typically used to treat OT can affect cerebellar function which might add information on the location of the oscillator. One study used positron emission tomography (PET) to show increased cerebellar blood flow bilaterally in OT subjects at rest. Moreover, bilateral cerebellar and contralateral lentiform and thalamic activation was present during postural upper limb tremor in OT patients, which was similar to previous findings in PET studies of patients with essential tremor and writing tremors [5,6]. A recent diffusion tensor imaging MRI study found white matter changes to be preferentially located in the cerebellum and in key components of the frontal-thalamic-cerebellar circuit [7] and MR spectroscopy showed a concordant NAA decrease in cerebellar white matter [8], while resting state MRI revealed a decrease of activity in cerebellar and somatosensory networks [9]. Damage to the cerebellum or its pathways has also been described in several cases of OT secondary to cerebellar cortical degeneration, pontine infarction [10,11,12], SCA2 [12], and SCA3 [13], all with associated limb ataxia.

Historically, OT has been mostly described as a pure tremor syndrome. In fact, there has been a paucity of prospectively collected data from large patient series specifically aimed at identifying accompanying signs and symptoms in primary OT patients. This might be in part due to the rarity of the condition. However, assessing patients in a systematic, controlled fashion has led to the discovery of other commonly associated signs and symptoms in OT, including cognitive dysfunction and anxiety-spectrum disorders [14,15]. In terms of presence of ataxia in OT, there is discrepancy in cohort descriptions ranging from a majority of patients with cerebellar symptoms in the only prospective study showing ataxia in 14 out of 18 examined subjects of a screened cohort of 37 patients [16], while retrospective analyses did not find ataxia at all in 68 OT patients [17] or exceptional cases of ataxia with 1 out of 5 patients designated as OT plus out of a cohort of 45 OT patients [18] and 2 patients out of 184 OT cases [19]. The lack of systematic and prospective data collection might be the culprit for mounting controversy [20,21,22].

Given the growing evidence of cerebellar involvement in OT, we hypothesized that ataxia may be more prevalent in this patient population than previously suggested by retrospective studies. This study attempts to answer the question of the prevalence of ataxic signs in patients with OT by the prospective, systematic performance of comprehensive ataxia testing in a relatively large cohort of OT participants.

## Methods

### Participants

This study was approved by the University of Nebraska Medical Center (UNMC) Institutional Review Board and was part of the UNMC Primary OT study, a prospective study with systematic assessments followed longitudinally [23]. Subjects from USA, Canada, Europe and Australia participated in the study. Initial informed consent was obtained over the phone due to subjects coming from large distances and live re-consent was obtained at the start of the study visits. Only participants with EMG-proven Primary OT with a 13–18 Hz tremor upon standing were included in the patient cohort, following the 1998 Consensus Statement on Tremors [2]. Their significant others were recruited as controls, which resulted in a male preponderance in the control group (65.0%) in contrast to the female preponderance in the OT group (87.9%). Exclusion criteria for both groups included known vestibular or balance problem or any other known diseases affecting movement. Cases of secondary OT were excluded. Evaluations were video recorded for the purpose of blinded reliability testing.

### Test Methods

The participants underwent a full neurological exam with a comprehensive ataxia assessment completed by movement disorder neurologists. The primary endpoint was a difference among the cohorts on the total score of the Brief Ataxia Rating Scale (BARS), a validated rating scale for ataxia. The BARS was scored in accordance with the parameters on the scale instructions [24]. Once the BARS was recorded, a semiquantitative coordination examination ensued, included detailed limb coordination testing (DLC) and detailed stance and gait testing (DS) (Table 1). Some of the tests (such as finger-to-nose) were performed twice (once for the BARS, and once for the DLC) and results reported as obtained. The upper and lower limb DLC exam was performed while sitting. Each component of the DLC and DS was scored by the specialists in a binary fashion (normal versus abnormal).

**Table 1:** Components of detailed limb coordination and detailed stance assessments.

Detailed limb coordination	Detailed stance

Finger to nose	Truncal sway or titubation while raising
Rapid alternating hand movements	Stance base
Finger chase	Stance with eyes closed and feet apart
Sequential finger movements	Stance with eyes open and feet together
Hand rhythm tapping	Stance with eyes closed and feet apart
Heel knee shin	Tandem gait
Toe following finger	Pull test

We understand that although the tests included in our DLC and DS evaluation have been historically used in clinical practice to ascertain coordination, their accuracy, sensitivity, specificity and reliability have not been systematically reported. This is why we chose the validated BARS as our primary measuring tool. And this is the reason why we also decided to use control subjects to increase the validity of the results. At the time of initial subject evaluation the neurological exam was videotaped. A subset of these video recordings was reevaluated by the investigators to assess reliability. To measure intra-rater reliability, each investigator rated subjects they had previously evaluated. To measure inter-rater reliability, two investigators rated subjects that they had not previously seen. The reliability testing was completed at one year from the original visits, and the investigators were blinded to the underlying diagnosis. A total of five investigators were involved in the inter-rater reliability process.

### Statistical Analysis

Significance was assessed by two-tailed Student’s t-test for BARS and Fisher’s Exact test for DLC and DS. Reliability was reported as percent agreement. The number of tests per participant varies slightly due to investigator’s omission error or the absence of good quality video recordings for the particular test.

## Results

### Demographics

34 OT subjects and 21 controls were enrolled. Sample characteristics can be seen in Table 2. The duration of symptoms for OT subjects ranged from 4 to 44 years (average 18) and average time to diagnosis from symptom onset was 7.7 years. A minority of the OT subjects were on medications for symptomatic treatment of their condition. These could potentially produce ataxic signs, and therefore have been listed on Table 2. None of our patients was taking more than one of these medications at the same time.

**Table 2:** Sample description.

	OT participants (range) N = 34	Healthy controls (range) N = 21

Age	66.5 (54–87)	66.1 (62–86)
Sex (% female)	87.9%	35%
Disease Duration in years	18 (4–44)	n/a
Time to diagnosis in years	7.7 (1–20)	n/a
**Relevant Medications**	**# of OT participants**	**# of controls**

Gabapentin	7	n/a
Clonazepam	4	n/a
Primidone	2	n/a
Levetiracetam, Pramipexole, Pregabalin, Propranolol, Topiramate	1	n/a

### Test results

**Figure 1:**
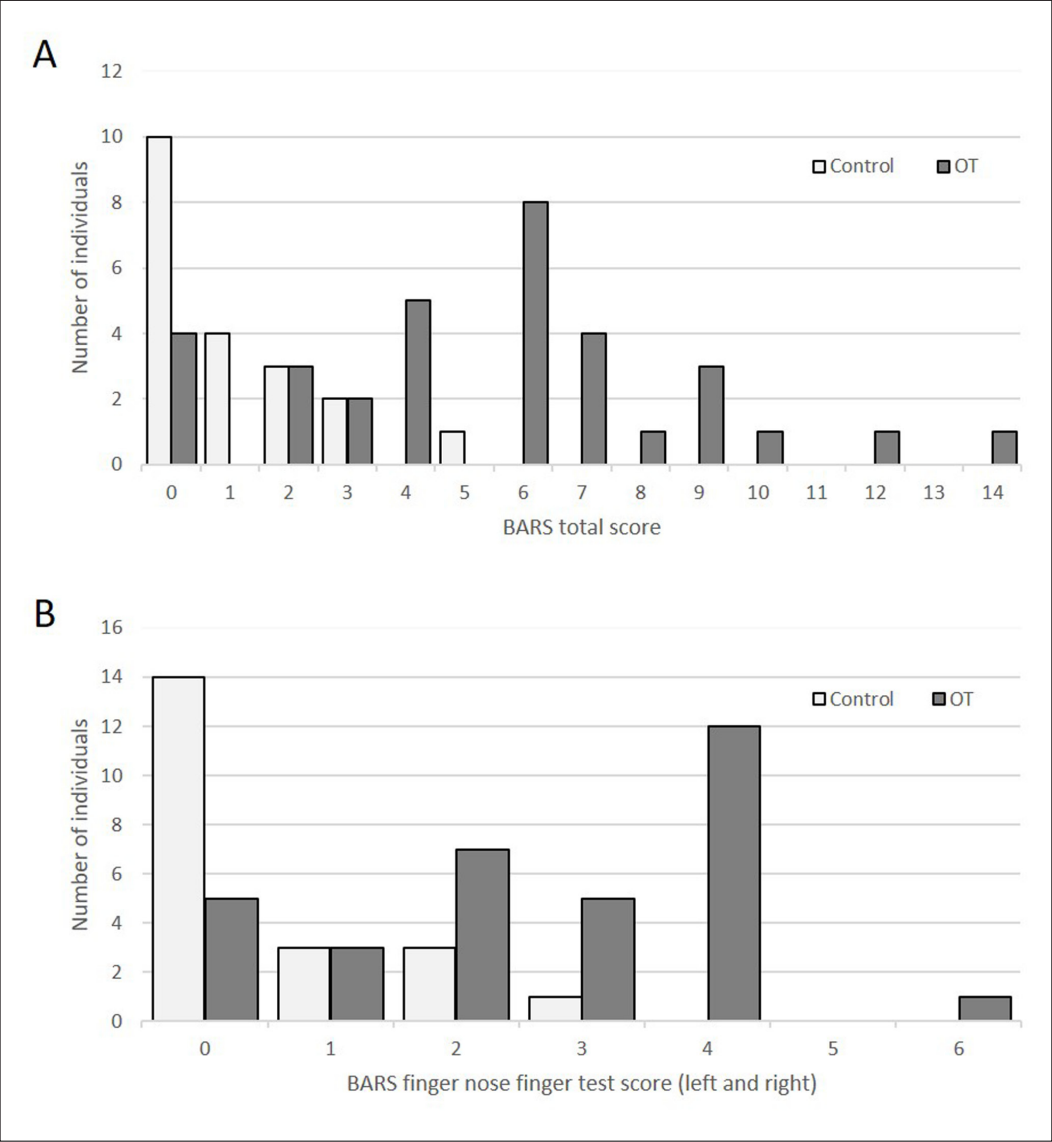
Distribution of BARS total scores **(A)** and BARS finger-to-nose-test scores **(B)** among OT patients versus controls.

There were statistically significant differences between OT patients and controls on the BARS. Using the most stringent criteria (BARS score equal or more than 1), twenty-four (87.5%) OT subjects had an abnormal score vs. 11 (52.4%) controls (Table 3). In 33 patients versus 21 controls BARS total score had a mean of 5.42 ± 3.40 versus 1.14 ± 1.42 (p < 0.001) and a median of 6 versus 1, respectively (Figure 1). When the scores were broken down, appendicular and truncal ataxia were found to be common in the OT subjects compared to controls. We did not find a significant difference in ocular or speech ataxia among our subjects (see Table 3). However, dysarthria and knee-tibia-test scores were elevated exclusively in the OT cohort, not in controls. We also found significant differences on the DLC and DS assessments which is consistent with what was found on the BARS (Table 3). There was no correlation between total BARS score and disease duration (data not shown).

**Table 3:** Coordination Exam results.

BRIEF ATAXIA RATING SCALE (n)	Number of patients with BARS item ≥1 (%)	Number of controls with BARS item ≥1 (%)	P Value (2-tailed Student’s t-test)

**Gait** (33 cases, 21 controls)	23 (69.7)	5 (23.8)	<0.01
**Knee tibia test – right** (33 cases, 21 controls)	11 (33.3)	0 (0)	0.01
**Knee tibia test – left** (33 cases, 21 controls)	8 (24.2)	0 (0)	0.03
**Finger to nose – right** (33 cases, 21 controls)	25 (85.8)	5 (23.8)	<0.01
**Finger to nose – left** (33 cases, 21 controls)	28 (84.8)	6 (28.6)	<0.01
**Dysarthria** (33 cases, 21 controls)	3 (9.1)	0 (0)	0.16
**Oculomotor** (33 cases, 21 controls)	13 (39.4)	6 (28.6)	0.34
**Number of Participants with BARS total ≥1** (33 cases, 21 controls)	29 (87.9)	11 (52.4)	<0.01
**BARS total Score (33 cases, 21 controls)**	**Median, Mean, SD**	**Median, Mean, SD**	**P Value (Chi Square Test)**

	6, 5.42, 3.16	1, 1.14, 1.42	<0.001
**DETAILED LIMB COORDINATION TESTING (n)**	**Number of patients with abnormal test (%)**	**Number of controls with abnormal test (%)**	**P Value (Fisher exact test)**

**Finger to nose** (34 cases, 21 controls)	24 (70.6)	8 (38.1)	<0.01
**Rapid alternating hand movements** (34 cases, 20 controls)	11 (32.4)	2 (10)	0.301
**Finger Chase (Finger following finger)** (34 cases, 20 controls)	28 (82.4)	8 (40)	<0.01
**Sequential hand movements** (34 cases, 21 controls)	21 (61.8)	4 (19)	<0.01
**Heel knee shin** (34 cases, 21 controls)	8 (23.5)	0 (0)	0.02
**Toe following finger** (34 cases, 20 controls)	23 (67.6)	7 (35)	0.01
**Hand rhythm tapping** (34 cases, 21 controls)	7 (21.2)	2 (9.5)	0.6
**DETAILED STANCE AND GAIT TESTING (n)**	**Number of patients with abnormal test (%)**	**Number of controls with abnormaltest (%)**	**P Value(Fisher exact test)**

**Truncal sway** (32 cases, 20 controls)	9 (28.1)	0 (0)	<0.01
**Stance base** (34 cases, 21 controls)	28 (82.4)	4 (19)	<0.01
**Stance with eyes closed feet apart** (34 cases, 20 controls)	25 (73.5)	0 (0)	<0.01
**Stance with eyes open and feet together** (34 cases, 19 controls)	26 (76.5)	2 (10.5)	<0.01
**Stance with eyes closed and feet together** (34 cases, 19 controls)	31 (91.2)	5 (26.3)	<0.01
**Tandem gait** (33 cases, 21 controls)	20 (60.6)	3 (14.3)	<0.01
**Pull test (more than 3 steps backwards)** (33 cases, 21 controls)	23 (69.7)	6 (28.6)	<0.01

### Intra- and Inter-rater Reliability Agreement

There was overall a very good level of reliability among our examiners in the performance of the many tests involved (Table 4). Intra-rater reliability for the DLC was 72.6% and inter-rater reliability 81.9%. Intra-rater reliability on the DS was 88% and inter-rater reliability 73.6%. An interesting finding was that alternating hand movements had 100% inter- and intra-rater reliability.

**Table 4:** Intra- and Inter-rater Reliability Testing Agreement.

Task	Intra-rater reliability agreement	Inter-rater reliability agreement

*Detailed Limb Coordination tasks*		
FTN	66.6%	79.2%
Rapid alternating hand movements	100%	100%
Finger Chase (finger following finger)	66.6%	79.2%
Sequential hand movements	66.6%	95.8%
Rhythm tap	75%	77.3%
Heel knee shin test	83.3%	91.7%
Toe following finger	50%	50%
Total Detailed Limb Coordination Score	72.6%	81.9%
*Detailed Stance tasks*		
Truncal sway	66.7%	70%
Raising up	83.3%	58.3%
Stance base	100%	91.7%
Stance with eyes closed and feet apart	83.3%	72.7%
Stance with eyes open and feet together	100%	54.5%
Stance with eyes closed and feet together	100%	54.5%
Tandem	83.3%	91.7%
Pull test	83%	91.7%
Total Detailed Stance Score	88%	73.6%

## Discussion

Based on this prospective study, the presence of ataxia in OT seems more common than previously reported [18,19]. Previous negative studies were either retrospective [18,19], or did not include systematic ataxia exams [25]. Our data is in agreement with previous findings of mild oculomotor ataxia found in prospective studies with distinct neuro-ophthalmologic methodology [16,26], while retrospective studies did find ataxia only in exceptional cases: no ataxia in 68 OT patients [17], 1 patient designated as OT plus with ET and ataxia among a cohort of 45 cases (5 OT plus) [18], 2 patients in a cohort of 184 patients [19]. The discrepancy could be in part due to disease duration, which was longer in the prospective study (14.1 years) and closer to our cohort’s (18 years), whereas the disease duration in retrospective studies was below 10 years [18,19]. One retrospective study however included follow up visits after a median of 6 years, which resulted in a disease duration of 14 years [17], but focused on progression of OT symptoms.

The strengths of our study include its prospective nature, the systematic assessment of ataxia, the use of validated scales, the use of multiple tests, the use of controls, the high level of intra- and inter-rater agreement, and the relatively large cohort for a rare disease. Limitations of our study include the relatively small cohort of patients. Also, a minority of our patients were taking medications for OT, which were not stopped for the sake of this study and could cause or worsen ataxia, particularly in toxic levels. But the use of toxic levels would be unlikely in these stable patients. In fact it has been reported that many OT patients end medical treatments quickly if side effects occur [20]. Importantly, many participants who were found to have signs of ataxia, were not taking any medications. Another important limitation is that by choosing to use spousal controls, the gender distribution was not egalitarian, particularly since more females are affected by OT.

The presence of ataxia or cerebellar dysfunction is germane to the pathophysiological understanding of many movement disorders and especially tremor disorders like essential tremor [27] and OT [28]. Ataxia was consistently mild in our sample, which renders its detection in OT more difficult. This might help explain why ataxia has not been consistently reported in OT. Importantly, ataxia scales have been primarily designed to rate severity of ataxia in known cerebellar disorders rather than as screening tools to extract mild ataxia from complex movement disorder patients. The difficulty of choosing an ataxia test in this setting is illustrated by a recent study where the authors were successfully using eyeblink classical conditioning as marker of cerebellar dysfunction in OT patients, while the clinical ataxia scale (SARA, Scale for the Assessment and Rating of Ataxia) [29] did not reach significance in their cohort of 13 patients compared to controls, most likely due to underpowered sample size [26]. As neurologists, we favor direct measurements of motor disability using clinical scales, which was the emphasis in our study. OT gives us the advantage of occurring only when standing, so that ataxia examinations carried out while sitting or lying down should be less affected by the tremor. However, we acknowledge that OT pathophysiology has not been unraveled yet and rare cases with symptoms in other body positions have been described [18]. A limitation of our study is that direct influence of subclinical tremor on BARS rating cannot be excluded completely. BARS does not contain any standing item as opposed to SARA [29] or ICARS [30], which might conveniently render it best for OT populations. We did not find a correlation of BARS total score and disease duration. However, since the OT-10 Severity Scale was not available when we completed the data collection, we were unable to calculate a correlation of OT and ataxia severity.

There is some agreement to a single central tremor generator in OT, but the location of such a generator remains unclear [28]. The findings of this study are supportive of cerebellar pathway involvement. Increased cerebellar activation seems undisputed in OT patients [6,31,32]. Moreover, activated cerebello-thalamic pathways seem to be involved in several tremor disorders such as essential and writing tremors [5]. It is unclear if cerebellar activation comprises an attempt to compensate tremor of a different source that may or may not arrive at attenuating it. Also a primary cerebellar defect could lead to OT oscillations based on recent findings of grey matter atrophy in cerebellar lobule VI with concurrent hypertrophy in the upper vermis and cerebrocortical motor regions in OT [31].

With our finding that ataxia is common in OT we see opportunities for additional studies. We did not evaluate brain images in our OT patients and in light of previous studies, cerebellar subregional volumes would be interesting to study in OT. Reproduction of previous studies of functional imaging [33] on a larger scale to enhance focal and time resolution would also be useful in determining the role of the cerebellum in OT. Also, as more findings are added to the list of motor and non-motor associations in primary OT (now to include ataxia, cognitive and behavioral components), revisions to existing diagnostic criteria might be entertained. Lastly, we believe that as clinicians, these findings could make us consider a more thorough coordination exam in patients with OT, and the avoidance of possible offending drugs.
